# Japanese Study on Stratification, Health, Income, and Neighborhood: Study Protocol and Profiles of Participants

**DOI:** 10.2188/jea.JE20130084

**Published:** 2014-07-05

**Authors:** Misato Takada, Naoki Kondo, Hideki Hashimoto

**Affiliations:** Department of Health and Social Behavior, University of Tokyo School of Public Health, Tokyo, Japan; 東京大学大学院医学系研究科 公共健康医学専攻 保健社会行動学

**Keywords:** Japan, socioeconomic status, social determinants of health, longitudinal studies

## Abstract

**Background:**

The Japanese Study on Stratification, Health, Income, and Neighborhood (J-SHINE) aims to clarify the complex associations between social factors and health from an interdisciplinary perspective and to provide a database for use in various health policy evaluations.

**Methods:**

J-SHINE is an ongoing longitudinal panel study of households of adults aged 25–50 years. The wave 1 survey was carried out in 2010 among adults randomly selected from the resident registry of four urban and suburban municipalities in the greater Tokyo metropolitan area, Japan. In 2011, surveys for the participants’ spouse/partner and child were additionally conducted. The wave 2 survey was conducted in 2012 for the wave 1 participants and will be followed by the wave 2 survey for spouse/partner and child in 2013.

**Results:**

Wave 1 sample sizes were 4357 for wave 1 participants (valid response rate: 31.3%; cooperation rate: 51.8%), 1873 for spouse/partner (response rate: 61.9%), and 1520 for child (response rate: 67.7%). Wave 2 captured 69.0% of wave 1 participants. Information gathered covered socio-demographics, household economy, self-reported health conditions and healthcare utilization, stress and psychological values, and developmental history. A subpopulation underwent physiological (*n* = 2468) and biomarker (*n* = 1205) measurements.

**Conclusions:**

Longitudinal survey data, including repeated measures of social factors evaluated based on theories and techniques of various disciplines, like J-SHINE, should contribute toward opening a web of causality for society and health, which may have important policy implications for recent global health promotion strategies such as the World Health Organization’s Social Determinants of Health approach and the second round of Japan’s Healthy Japan 21.

## Abstract (ja)

**背景：:**

まちと家族の健康調査（J-SHINE）は、学際的な見地から社会経済的要因と健康の複雑な関係を明確にし、様々な健康政策評価のためのデータベース提供を目的としたものである。

**方法：:**

まちと家族の健康調査は、25-50歳成人のいる世帯を対象としたパネル調査であり、現在進行中である。第1回調査は、東京近郊4市区の住民基本台帳よりランダム抽出した成人を対象として、2010年に実施した。さらに、参加者の配偶者・パートナーおよび子どもを対象とした調査を2011年に実施した。第2回調査は、第1回調査参加者に対して2012年に実施した。さらに、参加者の配偶者・パートナーおよび子どもに対する第2回調査を2013年に実施予定である。

**結果：:**

第1回調査の参加者は4,357名であった（有効回答率31.3％、協力率51.8％）。配偶者・パートナー調査には1,873名（有効回答率61.9％）、子ども調査には1,520世帯が参加した（有効回答率67.7％）。第2回調査の参加者は、第1回調査参加者の69.0％であった。調査内容には、社会人口統計学的要因、世帯経済状況、健康状態、医療利用状況、ストレス、心理的特性、発育歴を含めた。参加者の一部は、身体測定（2,468名）と血液検査（1,205名）にも参加した。

**結論：:**

まちと家族の健康調査のように、複数の学問分野における技法と理論に基づき、社会経済的要因を複数回測定する縦断的調査データは、社会と健康の複雑な因果関係の解明に必要である。また、世界保健機構による健康の社会格差に対する提言や日本における健康日本21（第二次）など、近年の世界的健康増進方略に対して重要な政策的含意を与えることが期待される。

## INTRODUCTION

Social determinants of health have become an important research topic in epidemiology. Previous studies have confirmed that poor health is unevenly distributed across socioeconomic positions, and findings from these studies have begun to be implemented in public health programs and measures worldwide.^1^ The World Health Organization has recommended that governments improve not only classic behavioral risks for health but also daily living conditions such as housing and neighborhood environments, and to “tackle the inequitable distribution of power, money, and resources.”^2^

However, several challenges face the social determinants of health studies, including: (1) the available evidence is mostly derived from studies using relatively simple data and methods, eg cross-sectional and cohort studies evaluating exposure at only a single time point; (2) the generalizability may be limited, as most of the theoretical and empirical evidence has been gathered in Western countries; and most importantly, (3) convincing theories that explain the mechanisms or pathways underlying the associations between social factors and health have yet to be proposed.^3^^,^^4^ To account for these challenges, hypotheses should be tested using trans- and inter-sectorial approaches in theory and methods, involving multiple disciplines such as economics, sociology, psychology, and molecular biology, as well as epidemiology and medicine. Longitudinal panel data are also necessary for conducting robust causal inferences accounting for the dynamic reciprocal interactions between society and health. However, such data are largely lacking, especially in non-Western societies.

Known examples of such studies are the Health and Retirement Survey in the United States and its sister projects worldwide, which are also available in Japan and other Asian nations.^5^^–^^8^ However, participants in these Health and Retirement Survey-family studies are limited to those aged 50 years or older. Large-scale epidemiological longitudinal studies have already been conducted in Japan, including the Japan Collaborative Cohort Study (JACC Study) and Japan Public Health Center-based Prospective Study (JPHC Study).^9^^–^^11^ These large cohort studies have contributed substantially to our understanding of the health consequences (eg, mortality and disease incidence) of behavioral and psychosocial risk factors at the individual level. More recently, these previous studies have provided some evidence of health disparity across education and occupational class, although their measurement of the economic and welfare conditions of households has been limited. Further, these studies relied fully or partially on visitors traveling to a health checkup at worksites or regional health centers for sampling.

In this regard, a population-based household survey with comprehensive measurement of social, economic, and health conditions would theoretically prove useful in tackling the complex mechanism through which social determinants in the household and their surrounding community exert influence on health. In addition, integration of multiple disciplines other than health sciences, including economics, sociology, community psychology, and policy sciences will be required to explore the wider social scientific interests in the interactive associations between social systems and individual health.

The purpose of the Japanese Study on Stratification, Health, Income, and Neighborhood (J-SHINE) is to provide an interdisciplinary, longitudinal survey database with comprehensive measures of living conditions, social environment, health, and biomarkers. With J-SHINE, we aim to understand (1) the current conditions for social stratification and their impacts on health disparities in Japan; (2) the biological, psychological, and social mechanisms of health disparities; (3) the impact of social and political systems on the development of social stratification and these factors’ roles in the control of health disparities; and (4) the possibility of promoting processes for social integration (eg, social capital) as tools to reduce health disparities. Here, we report the concept and designs of J-SHINE and the characteristics of its participants at baseline and in the following waves.

## METHODS

### Study design, setting, and participants

Adult community inhabitants, aged 25–50 years, were probabilistically selected from the residential registry in each of four municipalities (two in the Tokyo metropolitan area and two in neighboring prefectures). We intended to invite inner-sprawl urban and suburban regions, to account for variations in structural and social environments. For each municipality, 60 sample units were selected proportionally to the registered population, and systematic sampling^12^ was conducted for each unit, with oversampling of those aged in their 20s based on the expected lower response rate in this age stratum.

This sampling scheme, rather than a national representative random sampling, allowed us to collect data on individuals with various socioeconomic backgrounds under a homogeneous health, economic, and social policy environment, since each municipality forms a basic unit of local policy administration in Japan. With a sufficient number of samples, the scheme is expected to capture multilevel impact on health and related behaviors based on municipality characteristics and individual socioeconomic conditions. In addition, receiving endorsement from each municipality provides credibility which will help improve the response rate.

We contracted with independent survey agencies to conduct the surveys. Professional surveyors with more than three years of experience in conducting interview-based social surveys were recruited and underwent training sessions specifically to conduct the J-SHINE. The sessions lasted 6 hours for each wave, and included required lectures on the purpose of the survey, communication skills for home visiting and recruitment, contents of the questionnaire, operation of computer-based instruments and physiological measurement, and ethical consideration for confidentiality protection and safety, following training methods in previously established social surveys.^13^ We excluded poorly skilled surveyors during this training period. We further set up regular review sessions with surveyors during the survey wave in order to monitor their performance and quality of data collected, as well as to provide advice and consultation for troubleshooting.

The wave 1 survey was conducted between July 2010 and February 2011. The trained surveyors made at least five visits to reach the originally selected sample after sending an invitation letter. If they agreed to participate in the study, the participants were asked to provide written informed consent and then choose a convenient means for completing the survey questionnaire. Owing to the complex and contingent nature of the socioeconomic conditions among participants, we chose to use a computer-aided personal instrument (CAPI) to individually customize questionnaire items. The CAPI program was developed on an open-source platform and was accessible via the internet from the participant’s personal computer or on a left-behind laptop computer.

Surveyors provided participants with an ID and password for CAPI access and instructed participants on how to operate the CAPI session. The session was available around the clock and could freely be suspended and resumed at any point for participants’ convenience. Technical support was available by calling the support center. For those who were unfamiliar with computer use, a personal interview with the CAPI was provided. The collected data on left-behind computers were encrypted and sent via e-mail to our main server in the research laboratory. Each participant received a monetary incentive of ¥4000.

Among the participating households in the wave 1 survey, those with a spouse/partner of any age or child aged less than 18 years were invited to participate in supplemental surveys from August to December 2011. The spouse/partner survey asked the spouse/partner of the wave 1 participants to answer corresponding items to the wave 1 survey questionnaire to allow for pairwise comparisons. The child survey collected data on birth history and current conditions of child through the primary caregivers of the child, as well as through the child themselves if they were of school age. When the number of children was over three, the youngest three children were recruited for the survey.

**Figure 1.**
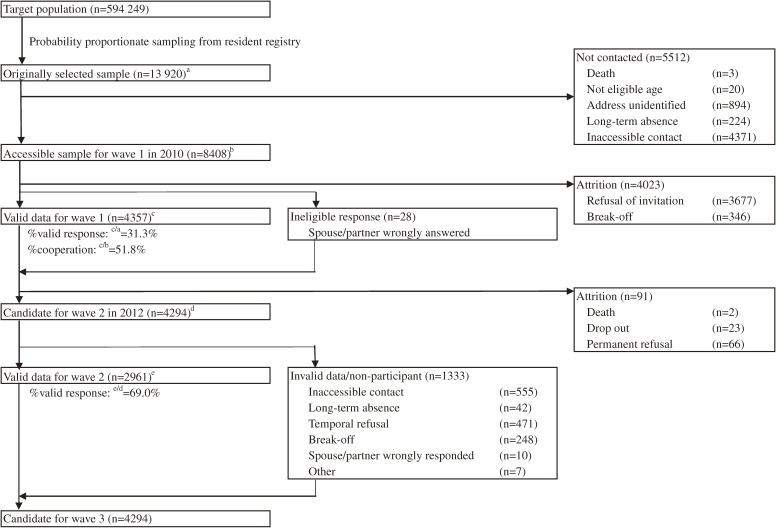
Flowchart of participant recruitment in J-SHINE. No contact: At least five visits by interviewers did not reach any members of the selected household. Long-term absence: The selected subject’s long-term leave was confirmed by the household’s co-residents. Drop out: Complete loss of follow-up information. Break-off: Less than 50% of the questionnaire items were completed.

**Figure 2.**
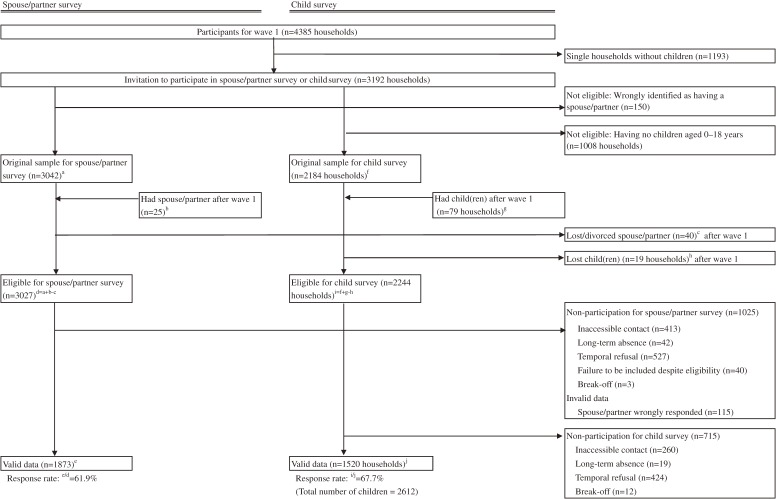
Flowchart of recruitment of participants for spouse/partner survey and child survey.

The wave 2 survey was conducted between July and December 2012. The participant recruitment is summarized in Figures 1 and 2. All of the questionnaires and measurement in the wave 2 survey were responded by the wave 1 participants.

### Measurements

The J-SHINE research team comprised multidisciplinary components, including sociology, economics, social psychology, cognitive science, health services, and social epidemiology. Each subspecialty group was asked to submit a set of questionnaire items relevant to addressing social determinants of health and their underlying mechanisms based on currently available theories and empirical studies. Among these, items regarded as commonly influential and applicable across disciplines were prioritized for selection.

Table 1 summarizes the adopted measures in the wave 1 survey, including: (1) demographic factors; (2) health and lifestyle; (3) medical insurance and utilization behaviors; (4) attitudes to health and life; (5) occupation and career path; (6) spouse/partner; (7) children; (8) parents; (9) social integration in the community; (10) job-related stress and psychology; (11) income, assets, and consumption; (12) social preferences; and (13) negative life events, including domestic violence, abuse, and suicidal events.

**Table 1.** List of data items collected in the J-SHINE wave 1 survey in 2010

**Demographic factors**	**Occupation and career**	**Social network**
**Health and lifestyle**	Current occupation	Bridging and bonding networks
Self-rated health	Working hours	Social support
Sleeping	Job demand and control	Social capital
Diet	First job	Years of residence at present address
Smoking	Education	**Occupational stress**
Alcohol consumption	Risk attitude	Job satisfaction
Physical activity	**Spouse/partner**	Work engagement
Height and weight	Education of spouse/partner	Work–life balance
Medical history	Occupation of spouse/partner	Effort–reward imbalance^©^ (10 item version)
Dental status	**Children**	**Income and expenses**
SF-8^©^	Demographics of children	Family head and dependent family members
Mental health (including K6)	Desired number of children	Monthly expenses (food, total)
**Medical insurance and consultation behaviors**	Expenses for child education/schooling	Annual income (individual, household)
Medical insurance	**Parents**	Home loans
Consultation use in the past 1 year	Current status of parents	Financial and other assets
Medical cost	Medical history of parents	Time preference
**Attitudes to health and life**	Education of parents	**Negative life events**
Happiness	Occupation of parents at respondent’s age 15	Traumatic event in the past 1 year
Stress coping	Household’s economy at respondent’s age 15	School bullying and absenteeism
Health literacy	Household’s economy at respondent’s age 5	Child abuse
Subjective socioeconomic status	**Cohabitation status and family doctors**	Domestic violence
Life satisfaction	Cohabitation status	Suicide attempt
Mastery	Family doctors	Sense of coherence

A full list of actual questions in the J-SHINE surveys are available online, URL: http://park.itc.u-tokyo.ac.jp/dhsb/project.html.

Questions asked in the spouse/partner survey were almost identical to those in the wave 1 survey, with items common in the household (eg, household size, year of marriage) excluded, as these values had already been obtained in the wave 1 survey.

Table 2 showcases the measures collected in the child survey. The CAPI for the primary caregivers requested information about each child on: (1) birth and vaccination history by referring to the maternity health record book; (2) maternity conditions, such as lifestyle and occupation status at pregnancy; and (3) child’s current health and lifestyles. Specifically, asthma and atopic dermatitis were diagnosed using the International Society of Asthma and Allergy in Childhood (ISAAC) battery.^14^^,^^15^ In cases of child aged less than 6 years, mother’s attachment and potential child abuse were inquired about using items from a questionnaire adopted by the Japan Environment and Children’s Survey.^16^^,^^17^ The primary caregivers were also asked to complete a paper-based survey on the child’s sociobehavioral development according to age (Denver II for age 0–9 months/10–23 months,^18^^,^^19^ Children Behavior Check List (CBCL) for age 2–3 years, and Strength and Difficulties Questionnaire (SDQ) for age 4 years and over).^20^^,^^21^ In addition, CBCL for ages 4–16 was used as an optional questionnaire.^22^^,^^23^ Child of school age were invited to answer a paper-based questionnaire evaluating lifestyles, daily time schedule, household cultural assets, and prospects for educational and occupational achievement. For those in junior high school and above, behaviors related to smoking, drinking, and sexual activities were also asked confidentially from their parents by sealing the questionnaires.

**Table 2.** Summary of data collected in the J-SHINE child survey in 2011

**Computer-based questionnaire for primary caregiver**	**Paper-based questionnaire for primary caregiver**
**Demographics**	**Child’s neurobehavioral development**
**Mother’s condition during pregnancy**	Denver II for age 0–9 months
Infertility treatment	Denver II for age 10–23 months
Work status before and after childbirth	Children Behavior Check List for age 2–3 years
Maternity leave	Children Behavior Check List for age 4 years or over
Lifestyle before and after childbirth	Strength and Difficulties Questionnaire for age 4 years or over
Health status before and after childbirth	**Paper-based self-administered questionnaire for school-age children**
Childcare support from family	**Health and lifestyle**
Childcare support from a spouse/partner	Current height and weight
Utilization of formal childcare services	Sleep schedule
Attachment (for those with a child aged 6 years or less)	Dietary habits
Abuse (for those with a child aged 6 years or less)	Oral hygiene
**Children’s health and lifestyle**	School life
Height (at birth, 1, 3, 6, 9, 18, and 36 months, current)	Time schedule
Weight (at birth, 1, 3, 6, 9, 18, and 36 months, current)	Learning experiences outside school hours
Health (at birth, 1, 3, 6, 9, 18, and 36 months, current)	Physical activity
Vaccination	Household chores
History of medical attention	Depression
Asthma (ISAAC battery)	Cultural assets available in the household
Atopic dermatitis (ISAAC battery)	Future careers and jobs
Sleep schedule	Smoking (for 12 years or over)
Dietary habits	Alcohol consumption (for 12 years or over)
Oral hygiene	Sexual behavior (for 12 years or over)

A full list of actual questions in the J-SHINE surveys are available online, URL: http://park.itc.u-tokyo.ac.jp/dhsb/project.html.

In the wave 2 survey, changes in the demographic, marital, occupational, economic, and health conditions were followed, with additional module questions on social preference (hypothetical dictator and ultimatum game) and social exclusion based on economic conditions. As an option, physiological measurements of blood pressure, respiratory peak flow, grip strength, and waist circumference were taken, and a paper-based dietary habits questionnaire^24^ was also completed. The participants were further invited to undergo blood chemistry measurements (LDL, HDL, HbA1c, triglyceride, high sensitivity C-reactive protein, and adiponectin) using a self-administered finger-prick blood sampling kit (Demecal Kit; Leisure Inc., Tokyo, Japan).

### Ethical issues

The study protocol and informed consent procedure were approved by the ethics committees of the Graduate School of Medicine of The University of Tokyo.

### Statistical analysis

All analyses were performed using the computer software SPSS 21.0J for Windows (IBM SPSS Japan Inc., Tokyo, Japan) and STATA11 for Windows (STATA Corp., College Station, TX, USA).

## RESULTS

### Main survey, wave 1

Of the 13 920 people in the originally selected sample, we were able to contact 8408 people for invitation to complete the interview survey (contact success rate: 60.4%). The reasons for not contacting subjects included death, out of eligible age range (25–50 years), moved, address unidentified, and long-term absence. Among the accessible sample, consent forms were submitted by 4731 participants, and valid responses were received from 4357 (2026 men and 2331 women; response rate: 31.3%; cooperation rate: 51.8%; Table 3). Of the participants, 3925 (90.0%) accessed the questionnaire through the internet, 412 (9.5%) used a stand-alone CAPI system, and 20 (0.5%) accepted an interview using the CAPI. Half of the participants who chose an interview were aged 45–50 years.

**Table 3.** Response rates and cooperation rates in the wave 1 survey by age and sex strata

	Men						Women					
	
Age (years)	25–29	30–34	35–39	40–44	45–50	Total	25–29	30–34	35–39	40–44	45–50	Total
No. of target population (2010 census based)^a^	54 232	61 751	73 439	66 070	65 289	320 781	51 011	57 326	67 765	59 835	59 660	295 597
No. of target population (registry base)	53 024	60 872	71 286	63 935	62 325	311 442	49 153	55 507	64 944	56 993	56 210	282 807
No. of originally selected sample (A)	1604^b^	1361	1504	1421	1281	7171	1609^c^	1250	1356	1261	1273	6749
No. of accessible sample (B)	809	762	861	855	791	4078	891	760	881	891	907	4330
No. of valid responses (C)	392	363	426	449	396	2026	457	415	493	487	479	2331

Valid response rate (C/A) (%)	24.4	26.7	28.3	31.6	30.9	28.3	28.4	33.2	36.4	38.6	37.6	34.5
Cooperation rate (C/B) (%)	48.5	47.6	49.5	52.5	50.1	49.7	51.3	54.6	56.0	54.7	52.8	53.8

Population share of census base (%)	17.0	19.5	22.9	20.6	20.0	100.0	17.4	19.6	23.0	20.1	19.9	100.0
Population share of valid respondents (%)	19.3	17.9	21.0	22.2	19.6	100.0	19.6	17.8	21.1	20.9	20.6	100.0

^a^Census 2010 available at http://www.stat.go.jp/data/kouhyou/e-stat_kokusei2010.xml.

^b^Includes 450 over-sampled participants.

^c^Includes 510 over-sampled participants.

The characteristics of the wave 1 participants are shown in Table 4. The obtained sample was fairly comparable with the vital statistics of the target population in terms of age and sex distribution, and percentages of graduates of high school or less in Census 2010 (data not shown in the table for education).

**Table 4.** Selected profiles of the participants for wave 1 and spouse/partner by sex

	Wave 1 survey		Spouse/partner survey	
	
	Men	Women	Men	Women
	(*n* = 2026)	(*n* = 2331)	(*n* = 958)	(*n* = 908)
	%	%	%	%
**Socioeconomic status**				
Education				
Junior high school	2.2	1.7	1.4	0.7
High school	23.8	24.0	21.2	26.4
Vocational school	15.2	18.0	13.4	18.9
Junior college	2.5	22.1	2.3	23.2
University	47.7	31.0	53.2	27.1
Postgraduate school	7.4	2.3	6.9	2.3
Missing	1.3	0.9	1.0	1.1
Work status				
Manager/executive	4.3	0.7	4.7	1.3
Regular employee	71.3	26.5	77.9	18.4
Contract/temporary/fixed-term employee	4.6	10.1	3.2	7.0
Part-time	5.2	25.4	1.4	25.0
Self-employed	6.9	5.7	9.1	6.5
Unemployment	2.4	1.7	0.9	3.1
Housekeeper	0.3	26.4	1.2	37.0
Student	1.6	0.5	0.2	0.3
Missing	0.8	0.5	0.7	0.7
Annual household income				
<2 million	3.5	3.4	—	—
2–5 million	19.7	18.7	—	—
5–7.5 million	22.4	19.2	—	—
7.5–10 million	16.8	15.5	—	—
≥10 million	13.5	13.1	—	—
Do not know	15.8	20.5	—	—
Missing	8.2	9.6	—	—
House ownership				
Own/spouse/partner	40.5	41.4	—	—
Parents	18.9	19.9	—	—
Missing	0.1	0.4	—	—
Subjective socioeconomic status				
High	2.5	2.0	4.9	3.6
Upper middle	22.5	23.0	30.7	30.7
Middle middle	32.9	38.3	38.0	36.0
Lower middle	26.9	22.2	15.7	14.9
Low	7.6	4.6	2.8	1.9
Do not know	6.9	9.5	7.3	12.6
Missing	0.7	0.5	0.6	0.3
**Social network**				
Number of communicating neighborhoods				
None	17.2	12.4	10.7	7.8
1–4	54.3	51.6	59.4	47.8
5–19	24.8	32.5	26.6	40.6
≥20	2.7	2.5	2.4	3.5
Having trust in one’s neighborhood	35.0	37.3	—	—
Number of close friends (mean ± SD)	7.0 ± 11.3	5.1 ± 6.4	—	—
**Health**				
Self-reported comorbidity				
Diabetes	1.6	0.6	1.9	0.4
Dyslipidemia	2.9	0.7	4.0	0.4
Depression/mental disorder	4.2	3.8	2.3	2.8
Hypertension	4.0	1.4	8.0	1.2
Asthma	1.5	2.3	1.8	2.5
Gastrointestinal complaint	0.8	1.0	0.9	1.0
Migraine	1.0	2.4	0.8	1.9
Cancer	0.2	0.7	0.2	0.4
Subjective health				
More than good	63.9	60.5	64.2	59.8
Missing	0.2	0.0	0.2	0.9
Body mass index				
≥25	27.0	10.1	25.6	7.3
Missing	1.3	5.3	0.9	5.1
SF-8^©^				
Physical component summary score (mean ± SD)	50.5 ± 6.3	49.6 ± 6.4	49.3 ± 6.4	50.1 ± 6.2
Mental component summary score (mean ± SD)	47.2 ± 7.4	47.2 ± 7.1	47.8 ± 6.9	47.9 ± 7.2
Missing	2.1	2.1	1.5	1.8
K6				
≥5	37.3	33.6	26.4	32.7
Missing	0.5	0.4	0.5	0.3
**Lifestyle**				
Current smoker	36.5	13.4	33.0	12.4
Ex-smoker	27.3	18.3	35.2	20.5
CAGE screening test for alcoholism ≥2	6.5	1.4	7.7	1.4
Exercise				
Every day	5.2	5.7	4.8	3.6
Seldom/never	58.4	61.9	61.8	70.6
Medical check-up ≥1/year	78.0	60.5	83.9	51.2
Private medical insurance subscriber	72.7	77.0	—	—
Having family doctors	68.1	78.2	—	—
**Family**				
Having a spouse/partner	65.9	72.1	—	—
Number of children				
0	47.2	38.7	—	—
1	18.5	19.9	—	—
2	25.5	30.0	—	—
≥3	8.0	10.9	—	—
Years of residence at present address (mean ± SD)	11.1 ± 10.8	11.1 ± 10.0	—	—

### Spouse/partner survey and child survey

From the results of the wave 1 survey, we identified 3027 participants who had spouse/partner eligible for our spouse/partner survey. Among these, 1991 families submitted a consent form for participating in the survey. Valid responses were received from 1873 spouses/partners (958 men, 908 women, and 7 unknown). Valid responses were provided for 61.9% of the initial candidates. The average age was 42.1 ± 7.8 years for male spouse/partner and 39.0 ± 6.3 years for female spouse/partner. The characteristics of the participants are shown in Table 4. Gender-specific composition of educational achievement and work status was comparable between wave 1 participants and spouse/partner survey, except that women in the spouse/partner survey were more likely to be homemakers.

Of the 2244 families eligible for the child survey, agreement forms were submitted by 1532 families, and valid responses were received from 1520 families (valid response rate: 67.7%) on 2612 children (1343 boys, 1257 girls, and 12 unknown). The age distribution of the participating child was as follows: 17.2% aged 0–3 years, 24.8% aged 4–6 years, 35.5% aged 7–12 years, and 22.5% aged 13–17 years, without significant differences between male and female sexes. Among those aged under 12 years, the 1-year prevalence of atopic dermatitis was 18.4% for boys and 18.8% for girls (*P* = 0.04), based on the ISAAC criteria (Table 5). The 1-year prevalence of asthma was 16.2% for boys and 11.7% for girls (*P* = 0.003). Regarding the children’s development measures, Denver II for children aged 0–9 months was obtained from 88 infants, Denver II for children aged 10–23 months from 195 toddlers, CBCL for children aged 2–3 years from 326 children, and SDQ for children aged 4 years or over from 1902 children. In the optional survey for primary caregivers, 1813 submitted CBCL for children aged 4 years or over.

**Table 5.** Selected profiles of the child survey by sex

	Boy (*n* = 1343)	Girl (*n* = 1257)
	*n* (%)	*n* (%)
Age		
0–3 years	230 (17.1)	218 (17.3)
4–6 years	342 (25.5)	303 (24.1)
7–12 years	473 (35.2)	449 (35.7)
13–17 years	298 (22.2)	287 (22.8)
Weight at birth (g, mean ± SD)	3056 ± 457	2965 ± 421
Subjective health		
More than good	923 (68.7)	884 (70.3)
Do not know	2 (0.1)	1 (0.1)
Missing	9 (0.7)	6 (0.5)
ISAAC for under 12 years child^a^		
Atopic dermatitis		
1-year prevalences	117 (18.4)	168 (18.8)
Current prevalences	114 (11.9)	120 (13.4)
Asthma		
life-time prevalences	309 (32.2)	219 (24.4)
1-year prevalences	156 (16.2)	105 (11.7)

^a^Denominator is number of under 12 years child: 961 boys, 896 girls.

ISAAC: International Study of Asthma and Allergies in Childhood.

For the self-administered questionnaire to children, 418 first through third graders at elementary school and 414 fourth through sixth graders at elementary school completed the questionnaire survey. Among 586 junior and senior high school children who responded to the questionnaire, 32 (5.5%) had ever smoked and 26 (5.5%) refused to answer about smoking habits. For drinking habits, 139 (23.7%) had ever drunk and 26 (4.4%) refused to answer the question. Finally, 31 (5.3%) responded that they had ever had sexual intercourse, 15 (2.6%) answered “do not know,” and 44 (7.5%) refused to answer.

### Main survey, wave 2

Of the 4294 people eligible for the wave 2 survey, we were unable to contact 597 (555 “no contact” and 42 “long-term absence”). Among 3219 who submitted consent forms, 248 break-offs and 10 ineligible observations were lost, resulting in 2961 valid responses (1309 men and 1652 women; response rate: 69.0%). Among them, 2825 also answered the dietary habits questionnaire, 2468 joined the physiological measurements, and 1205 further joined the blood chemistry checks. Table 6 exhibits nutrition intakes, estimated from the dietary habit questionnaire, and biomarker measurements. The average daily nutrition intake per energy intake (g/1000 kcal/day) of carbohydrate, protein, and fat were estimated higher among female participants compared to their male counterparts (*P* < 0.01 with *t*-test). The average systolic and diastolic blood pressures were significantly higher among males than among females (*P* < 0.01 with *t*-test). Average waist circumference of males was 88.1 ± 9.5 cm, while that of females was 78.8 ± 9.8 cm. Finally, average values of LDL cholesterol, HbA1c (in National Glycohemoglobin Standardization Project criteria), and adiponectin were all significantly higher among males (*P* < 0.01 with *t*-test).

**Table 6.** Selected profiles of the participant for wave 2 survey by sex

	Men	Women
	mean ± SD	mean ± SD
Daily nutrition intakes^a^		
Carbohydrate (g/1000 kcal/day)	132.4 ± 0.6	133.1 ± 0.5
Protein (g/1000 kcal/day)	34.0 ± 0.2	36.6 ± 0.1
Fat (g/1000 kcal/day)	27.8 ± 0.2	31.4 ± 0.2
Blood pressure^b^		
Systolic blood pressure (mm Hg)	124.6 ± 0.6	113.1 ± 0.4
Diastolic blood pressure (mm Hg)	79.5 ± 0.4	70.3 ± 0.3
Waist circumference (cm)^c^	88.1 ± 9.5	78.8 ± 9.8
Blood chemistry checks^d^		
LDL cholesterol (mg/dl)	107.7 ± 27.9	102.2 ± 31.7
HbA1c (%) (NGSP)^e^	5.5 ± 0.5	5.4 ± 0.4
Aadiponectin (µg/mL)	5.5 ± 3.0	9.5 ± 4.7

^a^Number of participant: 1231 men, 1594 women.

^b^Number of participant: 1036 men, 1397 women.

^c^Number of participant: 1052 men, 1410 women.

^d^Number of participant: 462 men, 743 women.

^e^National Glycohemoglobin Standardization Project.

## DISCUSSION

To our knowledge, J-SHINE is the most comprehensive household panel study thus far conducted in Japan, covering a wide range of participants’ lives during their life courses. This maximizes the capacities for examining the complex roles and interactions among macro-, meso-, and micro-social determinants of health. J-SHINE’s population-based sampling strategy also makes it possible to utilize external databases, such as census data, geo-spatial information, and other commercially available regional databases, that can be linked to the J-SHINE participants at the levels of community or zip codes for multilevel analysis.

The repeated measures of the comprehensive set of variables provide another important advantage, overcoming the major challenges of conventional cohort studies that cannot incorporate changes at the levels of exposure and misspecification owing to unmeasured confounders. These are specifically critical issues for causal inferences in studies of social determinants of health, as health both determines and is dynamically determined one’s income, occupation, and community roles. Given these advantages, J-SHINE may contribute to obtaining answers to some unique and politically important research questions, such as clarifying intergenerational causal pathways linking socioeconomic status and children’s health, the impact of community and government policies on childcare behaviors of parents and health of their children, couples’ joint decision-making on child-bearing and investment in childcare, and the health and socioeconomic consequences of irregular labor participation (Table 7).

**Table 7.** Examples of research questions that J-SHINE could be applied to

Child policies for households and their health impacts on children and parents
Intergenerational impacts of socioeconomic status on health
Time allocation (eg, share of working time vs leisure time) and household’s production function of health
Health impacts of working conditions (eg, permanent vs precarious, wage differential, availability of worker’s compensation, etc) and spillover to partner/children
Contagion of health and health behaviors among partners/families
Behavioral economic study (eg, time preference) on health behavior
Biological responses to social stresses
Bio-psychological mechanisms of health behavioral choices

The wave 2 survey in 2012 will be succeeded by follow-up studies of spouse/partner and child of the wave 1 participants. We intend to continue follow-up of the family as a whole at least until the included children reach high school graduation in order to clarify how social environments and health conditions interactively affect children’s well-being in early adulthood, as long as sufficient funding allows.

Two major limitations of J-SHINE warrant mention. First, despite the remarkable advantages attributable to our sampling strategy, the external validity for the entire Japanese population is questionable because the data are not nationally representative and the response rate is relatively low. However, we confirmed that the distribution of the demographic characteristics of the J-SHINE participants is representative of the targeted municipality residents. Second, using mortality data as a “hard” health outcome is unrealistic in J-SHINE, as the majority of its participants are young and the mortality incidence is very low thus far. Instead, we designed J-SHINE to explore the biological and social pathways and mechanisms linking social systems and intermediate health risks such as behaviors, psychological response, and biomarkers.

In conclusion, we believe that a comprehensive interdisciplinary study like J-SHINE is necessary to open a web of causality for the associations between society and health and to advance recent public health targets of tackling social determinants of health and reducing health disparities, as outlined in the WHO’s initiative policy.^1^^,^^25^ In Japan, the new Healthy Japan 21 (*in Japanese: kenko nihon 21 – dainiji*) has added “reducing health disparities” as one of its two overall goals (with the other being “extension of healthy longevity”) for its second round since 2012.^26^

We plan to make the J-SHINE data open access for research purposes on an application and approval basis in the near future. Although the J-SHINE data can be used in many disciplines, including those other than health, such as economics and sociology, the most useful way to employ the data may be analyses with interdisciplinary perspectives. When carrying out analyses, caution is needed in the interpretation of the variables measured in J-SHINE, as some variables require understanding of specific theories and techniques for use in a discipline.

## ONLINE ONLY MATERIALS

Abstract in Japanese.
