# Supplementary material for: Japanese Study on Stratification, Health, Income, and Neighborhood: Study Protocol and Profiles of Participants
Source: J Epidemiol. 2014 Jul 5;24(4):334–44. doi: 10.2188/jea.JE20130084 (PMC4074639; doi:10.2188/jea.JE20130084)
Supplement: Abstract in Japanese. [file je-24-334-s001.pdf]

まちと家族の健康調査（J-SHINE）：調査概要

髙田未里（東京大学大学院医学系研究科　公共健康医学専攻　保健社会行動学）
近藤尚己（東京大学大学院医学系研究科　公共健康医学専攻　保健社会行動学）
橋本英樹（東京大学大学院医学系研究科　公共健康医学専攻　保健社会行動学）

背景：まちと家族の健康調査（J-SHINE）は、学際的な見地から社会経済的要因と健康の複雑な関係を明確にし、様々な健康政策評価のためのデータベース提供を目的としたものである。
方法：まちと家族の健康調査は、25-50 歳成人のいる世帯を対象としたパネル調査であり、現在進行中である。第 1 回調査は、東京近郊 4 市区の住民基本台帳よりランダム抽出した成人を対象として、2010 年に実施した。さらに、参加者の配偶者・パートナーおよび子どもを対象とした調査を 2011 年に実施した。第 2 回調査は、第 1 回調査参加者に対して 2012 年に実施した。さらに、参加者の配偶者・パートナーおよび子どもに対する第 2 回調査を 2013 年に実施予定である。
結果：第 1 回調査の参加者は 4,357 名であった（有効回答率 31.3%、協力率 51.8%）。配偶者・パートナー調査には 1,873 名（有効回答率 61.9%）、子ども調査には 1,520 世帯が参加した（有効回答率 67.7%）。第 2 回調査の参加者は、第 1 回調査参加者の 69.0%であった。調査内容には、社会人口統計学的要因、世帯経済状況、健康状態、医療利用状況、ストレス、心理的特性、発育歴を含めた。参加者の一部は、身体測定（2,468 名）と血液検査（1,205 名）にも参加した。
結論：まちと家族の健康調査のように、複数の学問分野における技法と理論に基づき、社会経済的要因を複数回測定する縦断的調査データは、社会と健康の複雑な因果関係の解明に必要である。また、世界保健機構による健康の社会格差に対する提言や日本における健康日本 21（第二次）など、近年の世界的健康増進方略に対して重要な政策的含意を与えることが期待される。

キーワード
日本、社会経済的状態、健康の社会格差、縦断研究
